# HDAC inhibitor Trichostatin A suppresses adipogenesis in 3T3-L1 preadipocytes 

**DOI:** 10.18632/aging.203238

**Published:** 2021-07-07

**Authors:** Xin Lv, Jun Qiu, Tao Hao, Haoran Zhang, Haiping Jiang, Yang Tan

**Affiliations:** 1 Department of Clinical Nutrition, The First Affiliated Hospital of Jinan University, Guangzhou 510630, Guangdong, China; 2 Guangzhou Key Laboratory of Molecular and Functional Imaging for Clinical Translation, The First Affiliated Hospital of Jinan University, Guangzhou 510630, Guangdong, China; 3 Department of General Surgery, The First Affiliated Hospital of Jinan University, Guangzhou 510630, Guangdong, China; 4 Department of Pathology, The Seventh Affiliated Hospital of Sun Yat-Sen University, Shenzhen 518000, Guangdong, China

**Keywords:** Trichostatin A, obesity, adipogenesis, PPARγ, AMPK

## Abstract

Background and purpose: Obesity is becoming a major global health issue and is mainly induced by the accumulation of adipose tissues mediated by adipogenesis, which is reported to be regulated by peroxisome proliferator-activated receptor γ (PPARγ) and CCAAT enhancer-binding protein α (C/EBPα). Trichostatin A (TSA) is a novel histone deacetylase inhibitor (HDACI) that was recently reported to exert multiple pharmacological functions. The present study will investigate the inhibitory effect of TSA on adipogenesis, as well as the underlying mechanism.

Methods: The adipogenesis of 3T3-L1 cells was induced by stimulation with a differentiation cocktail (DMI) medium for 8 days. MTT assay was used to measure the cell viability and Oil Red O staining was used to evaluate the adipogenesis of 3T3-L1 cells. The total level of triglyceride and released glycerol were detected to evaluate the lipolysis during 3T3-L1 adipogenesis. The expression levels of *Leptin*, fatty acid-binding *protein 4 (FABP4)*, and *sterol regulatory element-binding protein* (*SREBP1C)* were determined by qRT-PCR. qRT-PCR assay was utilized to detect the expression levels of PPARγ and C/EBPα in 3T3-L1 cells. A high-fat diet (HFD) was used to construct an obese mice model, followed by the treatment with TSA. HE staining was conducted to evaluate the pathological state of adipose tissues. Body weights and the weights of adipose tissues were recorded to evaluate the anti-obesity property of TSA.

Results: Firstly, the promoted lipid accumulation induced by DMI incubation was significantly reversed by the treatment with TSA in a dose-dependent manner. The elevated expression levels of *Leptin*, *FABP4*, *SREBP1C,* PPARγ, and C/EBPα induced by the stimulation with DMI incubation were dramatically inhibited by the introduction of TSA, accompanied by the upregulation of phosphorylated AMP-activated protein kinase (p-AMPK). Secondly, the inhibitory effect of TSA against the expression level of PPARγ and lipid accumulation was greatly abolished by an AMPK inhibitor. Lastly, the increased body weights and visceral adipocyte tissue weight, as well as the enlarged size of adipocytes induced by HFD were pronouncedly reversed by the administration of TSA.

Conclusion: TSA inhibited adipogenesis in 3T3-L1 preadipocytes by activating the AMPK pathway.

## INTRODUCTION

Obesity is a complex chronic disease caused by multiple elements, and is characterized by excessive accumulation of lipids. Obesity is often accompanied by life-threatening diseases, such as atherosclerosis, hyperlipidemia, fatty liver, diabetes, cancer, etc [1, 2]. In our body, excessive ingested energy is stocked in adipose tissue in the form of fat, which is discomposed to fatty acid, released into the blood and used as an energy supply for other tissues in the absence of external energy supply [3]. Therefore, adipose tissues are regarded as important organs for energy storage and important regulators for maintaining the energy balance and glucose homeostasis [4]. However, obesity develops when adipose tissue accumulates excessively. The obesity phenotype is mainly induced by the transformation from pre-adipocytes to adipocytes due to the enlargement and proliferation of adipose tissues [5]. The conversion of preadipocytes to adipocytes is mediated by a series of key transcriptional factors, including peroxisome proliferator-activated receptor γ (PPARγ) and CCAAT enhancer-binding protein α (C/EBPα), which regulate the expressions of multiple lipid proteins [6, 7]. PPARγ is classified into three subtypes: PPARγ1, PPARγ2, and PPARγ3. PPARγ1 is lowly expressed in most tissues and PPARγ3 is mainly expressed in macrophages and large intestine cells [8]. PPARγ2 is mainly expressed in adipose tissues and is reported to be involved in the advanced stage of adipocyte differentiation [9]. The significance of PPARγ in adipocytes was verified in PPARγ knockdown mice, which were found dead several days after maturation [10]. When PPARγ is activated, a dimer is formed through the binding between PPARγ and its retinoic acid receptor, further regulating the transcription of its downstream target genes by interacting with the specific peroxisome proliferator responsive element (PPRE) of these target genes, including fatty acid-binding protein 4 (FABP4) and sterol regulatory element-binding protein 1c (SREBP1c) [11]. C/EBP is the first discovered transcriptional factor that plays an important role in the process of adipocytes differentiation, it is classified into 6 isomers. In the differentiation process of 3T3-L1 preadipocytes, C/EBPα, C/EBPβ, and C/EBPδ are the main functional isomers. In the early stage of adipocytes differentiation, C/EBPβ, and C/EBPδ are significantly upregulated by stimulation of hormones, further activating the expressions of PPARγ and C/EBPα to induce the differentiation of adipocytes [12]. Therefore, upregulation of PPARγ and C/EBPα is observed in the advanced stage of adipocyte differentiation and might be an effective target for the treatment of obesity.

Trichostatin A (TSA) is a novel histone deacetylase inhibitor (HDACI) that belongs to the isohydroxamic acid category. TSA suppresses the activity of histone deacetylase by binding to the zinc ionic bond at the bottom of the tubular structure of histone deacetylase with its isohydroxamic acid ligand [13]. TSA is reported to exert many pharmacological functions, such as anti-inflammation [14], anti-tumor property [15], and neuroprotective effects [16]. Recently, inactivation effects on PPARγ [17] and C/EBPα [18] have been reported. In the present study, the inhibitory effect of TSA on adipocyte differentiation, as well as the underlying mechanism, will be investigated to explore the potential therapeutic property of TSA on obesity.

## RESULTS

### The effects of TSA on cell viability of 3T3-L1 cells

**Figure 1:**
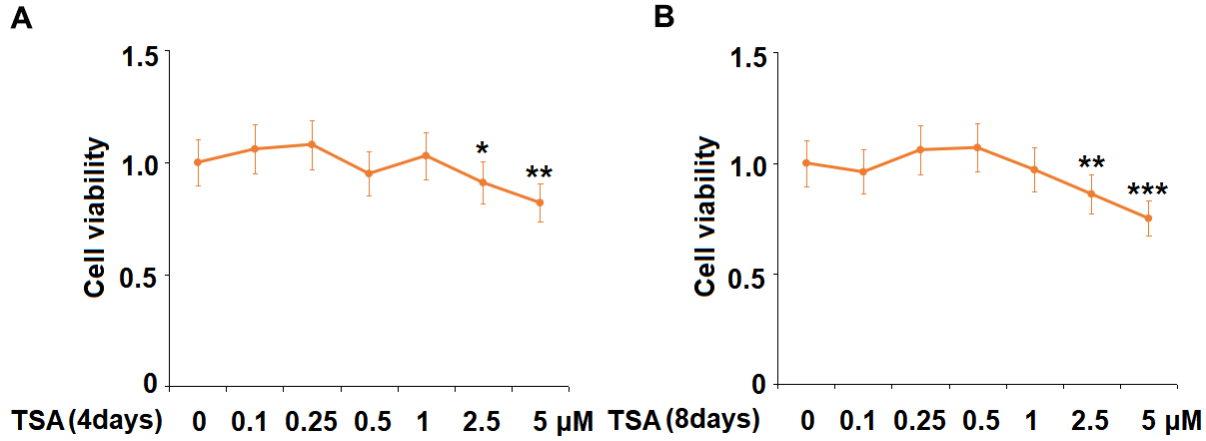
**The effects of TSA on cell viability of 3T3-L1 cells.** Cells were stimulated with 0.1, 0.25, 0.5, 1, 2.5, and 5 μM TSA for 4 days and 8 days. (**A**) Cell viability was determined using an MTT on day 4; (**B**) Cell viability was determined with MTT on day 8 (n= 5-6, *, **, ***, P<0.05, 0.01, 0.001 vs. vehicle group).

Cells were stimulated with 0.1, 0.25, 0.5, 1, 2.5, and 5 μM TSA for 4 days and 8 days, followed by evaluating the cell viability with an MTT assay. As shown in Figure 1, on both Day 4 and 8, no significant difference in the cell viability was observed as the concentration of TSA increased from 0.1 to 1 μM. However, the cell viability decreased significantly as the concentration of TSA increased to 2.5 and 5 μM. Therefore, 0.5 and 1 μM were utilized in the subsequent experiments.

### TSA inhibited adipogenesis of 3T3-L1 cells

**Figure 2:**
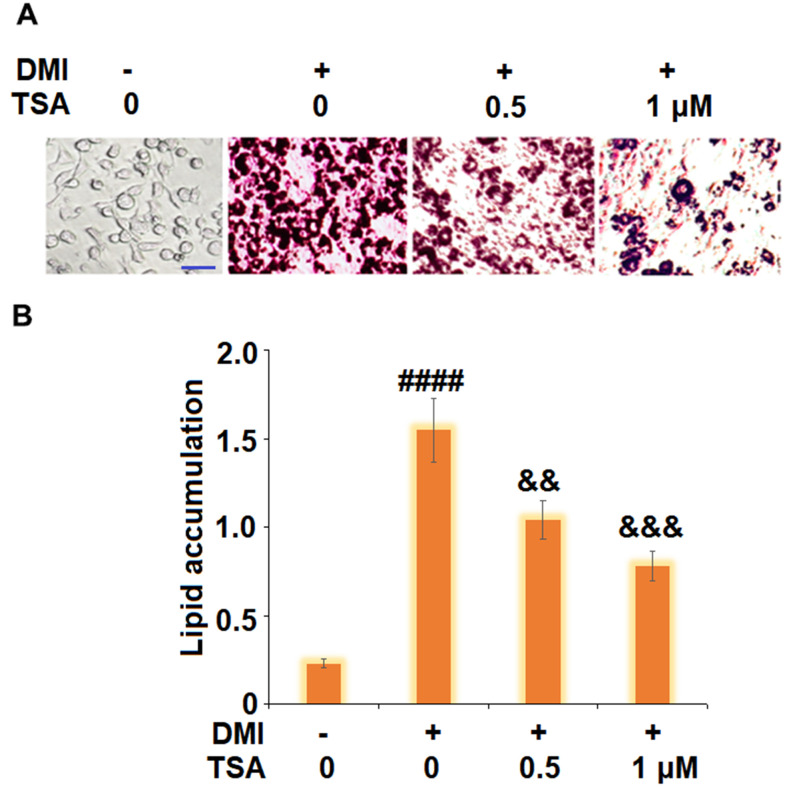
**TSA inhibits adipogenesis of 3T3-L1 cells.** Cells were incubated with a differentiation cocktail (DMI) medium with TSA (0.5 and 1 μM). (**A**) Cells were stained with Oil Red O on day 8; (**B**) Lipid accumulation was examined by measuring absorbance at 540 nm of Oil Red O staining. Scale bar, 100 μm (n=5-6, ####, P<0.0001 vs. vehicle group; &&, &&&, P<0.01, 0.001 vs. DMI treatment group).

To evaluate the impact of TSA on adipogenesis in 3T3-L1 cells, cells were incubated with a differentiation cocktail (DMI) medium with TSA (0.5 and 1 μM). As shown in Figure 2, the lipid accumulation was significantly elevated by stimulation with DMI, but was dramatically suppressed by the introduction of TSA in a dose-dependent manner.

### TSA promoted lipolysis during 3T3-L1 adipogenesis

**Figure 3:**
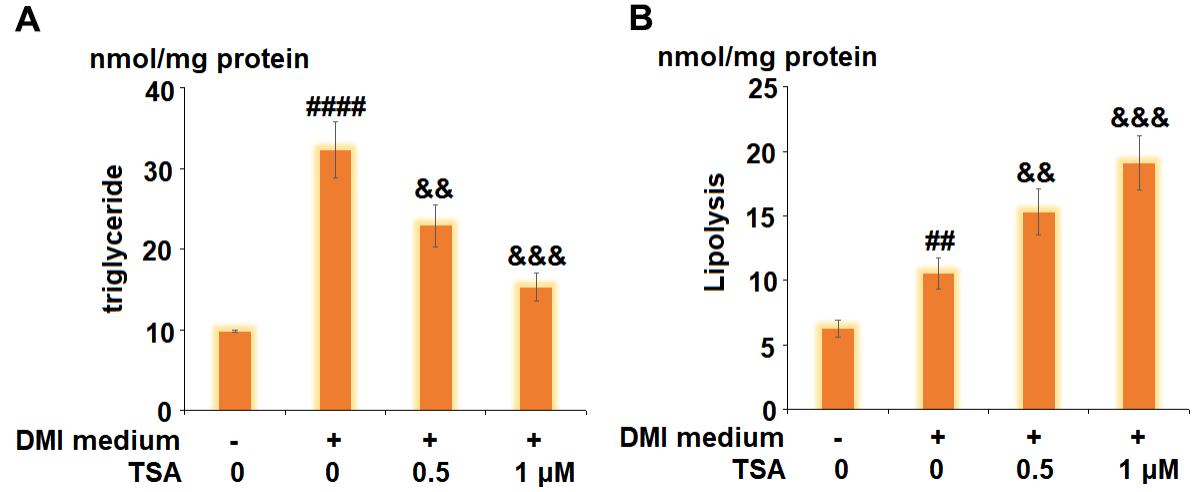
**TSA promotes lipolysis during 3T3-L1 adipogenesis.** Cells were incubated with differentiation cocktail (DMI) medium with TSA (0.5 and 1 μM). (**A**) Total level of triglyceride; (**B**) Lipolysis is shown as glycerol release (n=5-6, ##, ####, P<0.01, 0.0001, VS. vehicle group; &&, &&&, P<0.01, 0.001 vs. DMI treatment group).

The levels of triglyceride and glycerol were detected to evaluate the lipolysis of 3T3-L1 cells. As shown in Figure 3A, compared to the control, the level of triglyceride was increased from 9.8 nmol/mg protein to 32.3 nmol/mg protein by stimulation with DMI but was inhibited to 22.9 and 15.3 nmol/mg protein by treatment with 0.5 and 1 μM TSA, respectively. In addition, the release of glycerol was elevated from 6.3 nmol/mg protein to 10.5 nmol/mg protein by the treatment with DMI but was decreased to 15.3 and 19.1 nmol/mg protein by the introduction of 0.5 and 1 μM TSA, respectively. These data indicate that the lipolysis during 3T3-L1 adipogenesis was promoted by TSA.

### TSA suppressed the expression of *Leptin*, *FABP4*, and *SREBP1C* during 3T3-L1 adipogenesis

**Figure 4:**
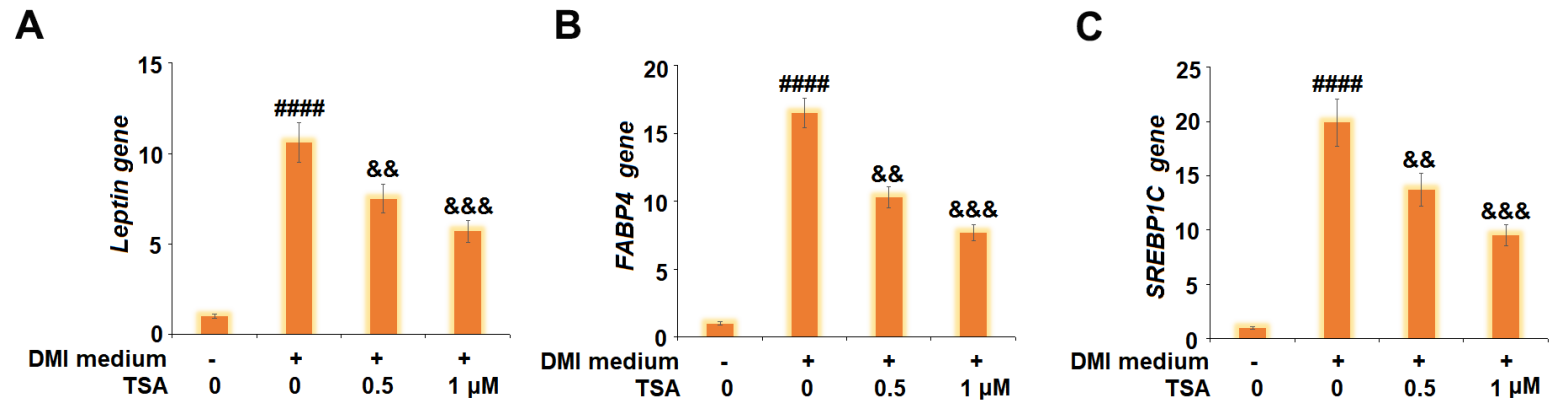
**TSA suppresses the expression of *Leptin*, *FABP4*, and *SREBP1C* during 3T3-L1 adipogenesis.** Cells were incubated with a differentiation cocktail (DMI) medium with TSA (0.5 and 1 μM). (**A**) mRNA of *Leptin;* (**B**) mRNA of *FABP4;* (**C**) mRNA of *SREBP1C* (n=5-6, ####, P<0.0001 vs. vehicle group; &&, &&&, P<0.01, 0.001 vs. DMI treatment group).

We further measured the expression level of adipogenesis-related genes. As shown in Figure 4, the gene expression levels of *Leptin*, *FABP4*, and *SREBP1C* were significantly promoted by incubation with DMI and greatly suppressed by the introduction of TSA. These data indicate that the process of 3T3-L1 adipogenesis might be suppressed by TSA.

### TSA suppressed the expression of adipogenic and lipogenic transcriptional factors in 3T3-L1 cells

**Figure 5:**
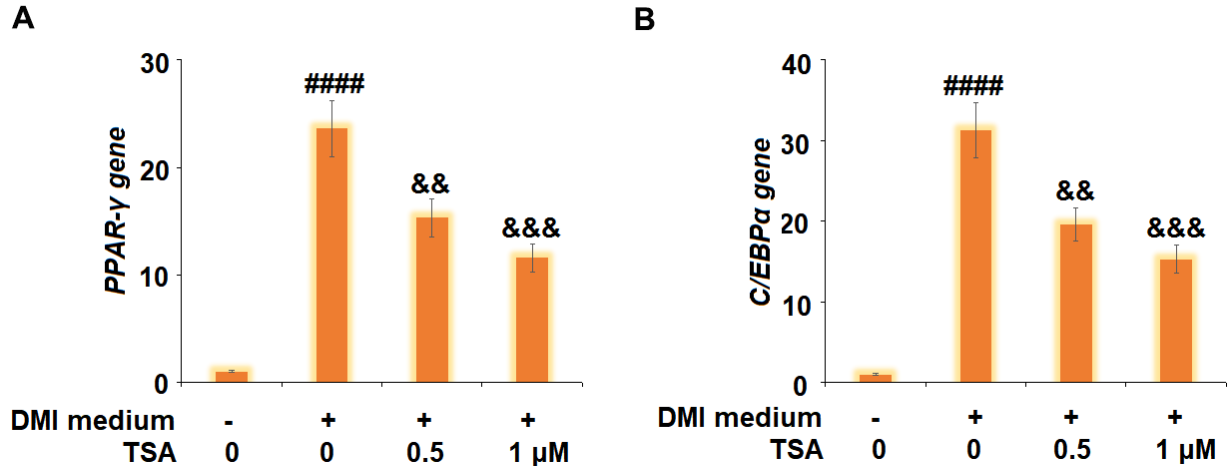
**TSA suppresses the expression of adipogenic and lipogenic transcriptional factors in 3T3-L1 cells.** Cells were incubated with a differentiation cocktail (DMI) medium with TSA (0.5 and 1 μM). (**A**) mRNA of PPAR-γ; (**B**) mRNA of C/EBPα (n=5-6, ####, P<0.0001 vs. vehicle group; &&, &&&, P<0.01, 0.001 vs. DMI treatment group).

The gene and protein expression levels of PPAR-γ and C/EBPα were subsequently evaluated. As shown in Figure 5, we found that PPAR-γ and C/EBPα were significantly upregulated by DMI incubation and later downregulated by treatment with TSA. These data indicate that the expressions of adipogenic and lipogenic transcriptional factors in 3T3-L1 cells induced by DMI were pronouncedly inhibited by TSA.

### TSA inhibited adipogenesis by activating the AMPK pathway

**Figure 6:**
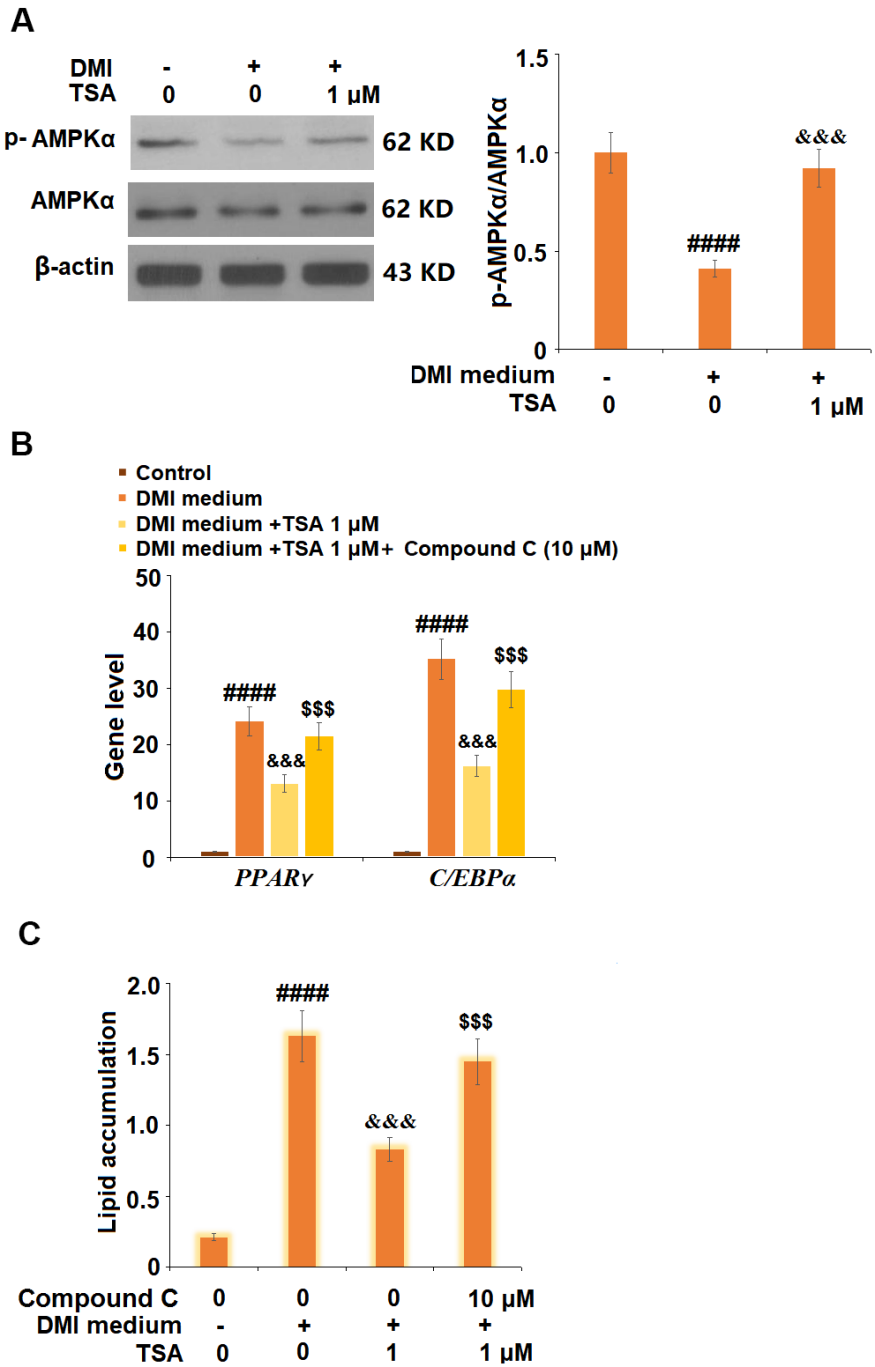
**TSA activates the AMPK pathway.** (**A**) Cells were incubated with a differentiation cocktail (DMI) medium with TSA (1 μM). Phosphorylated and total levels of AMPKα were measured; (**B**) Cells were incubated with differentiation cocktail (DMI) medium with TSA (1 μM) or Compound C (10 μM) for 8 days. mRNA of *PPAR-γ* and C/EBPα; (**C**) Lipid accumulation was examined by measuring absorbance at 540 nm of Oil Red O (n=5-6, ####, P<0.0001 vs. vehicle group; &&&, P< 0.001 vs. DMI treatment group; $$$, P<0.001 vs. DMI+TSA group).

To explore the potential mechanism, cells were incubated with a differentiation cocktail (DMI) medium with TSA (1 μM). The expression of p-AMPKα was significantly suppressed by treatment with DMI but dramatically elevated by the introduction of TSA. Subsequently, 3T3-L1 cells were incubated with differentiation cocktail (DMI) medium with TSA (1 μM) or Compound C (10 μM), an inhibitor of the AMPK pathway, for 8 days. As shown in Figure 6B, the elevated expression level of PPAR-γ induced by DMI was significantly inhibited by treatment with TSA, and further promoted by the co-incubation with Compound C. In addition, the increased lipid accumulation (Figure 6C) in 3T3-L1 cells induced with DMI was greatly suppressed by treatment with TSA and further elevated by the co-incubation with Compound C. These data indicate that the bio function of TSA might be mediated by the activation of the AMPK pathway.

### TSA reduced the weight of visceral adipocyte tissue and the bodyweight of HFD-fed obese mice

**Figure 7:**
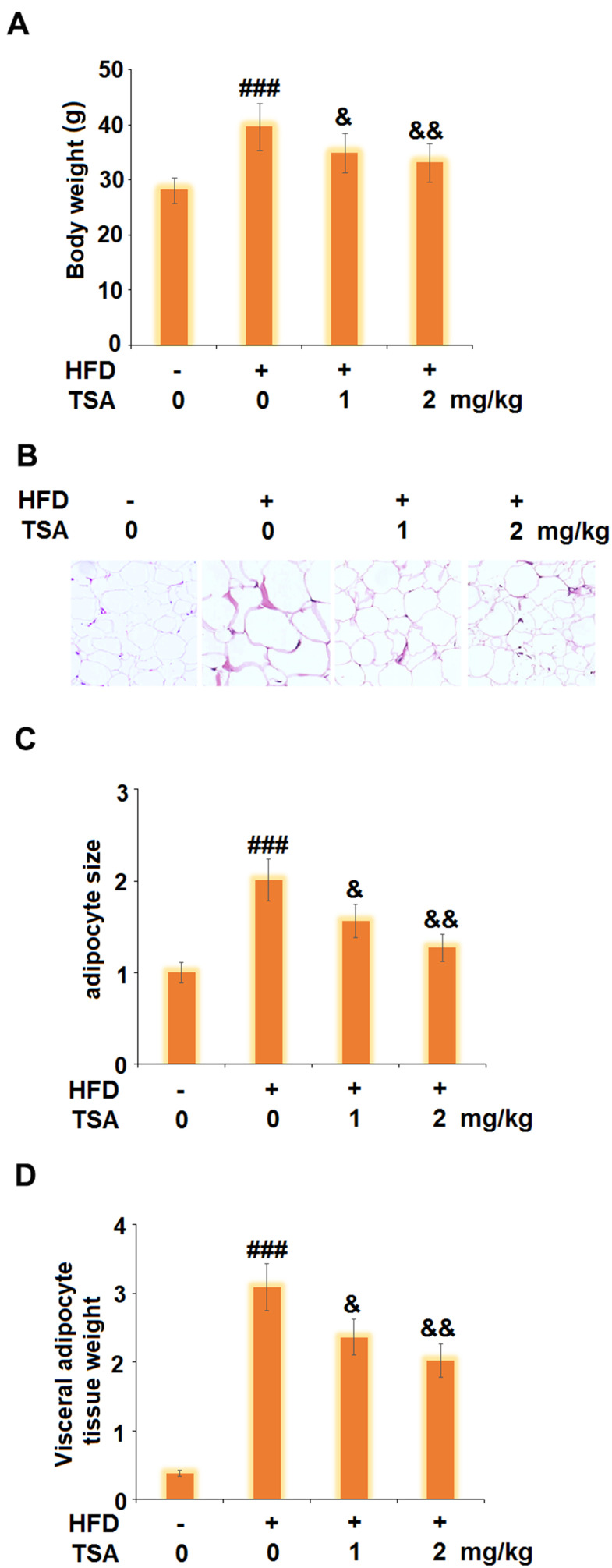
**TSA reduces the weight of visceral adipocyte tissue and the bodyweight of HFD-induced obese mice (10 weeks, start with 6-8 weeks old B6 male mice).** (**A**) Bodyweight; (**B**) Histological sections of visceral adipocyte tissue; (**C**) Quantification of adipocyte size; (**D**) Visceral adipocyte tissue weight (n=5-6, ###, P<0.001 vs. vehicle group; &, &&, P<0.05, 0.01 vs. HFD group).

To verify the inhibitory effect of TSA against obesity, the obese mice were established followed by consecutive treatment with TSA. As shown in Figure 7A, compared to the control, the body weight of mice in the HFD group was elevated from 28.1 g to 39.5 g and suppressed to 34.9 g and 33.1 g after the administration of 1 mg/kg and 2 mg/kg TSA, respectively. HE staining images (Figures 7B, 7C) indicated that compared to the control, an enlarged size of adipocytes was observed in the HFD group but significantly alleviated by treatment with 1 mg/kg and 2 mg/kg TSA. In addition, as shown in Figure 7D, significantly higher visceral adipose tissue weight was observed in the HFD group which was decreased in the 1 mg/kg and 2 mg/kg TSA groups. These data verified the inhibitory property of TSA against the HFD-induced obesity in mice.

## DISCUSSION

With the upturn in living standards, obesity has become a global health issue due to irregular lifestyles and eating habits. At the cellular level, obesity is mainly caused by the increase in size and number of adipocytes [19], originating from the transformation of preadipocytes. The transformation from preadipocytes to mature adipocytes is mediated by several stimulators, such as glucocorticoids, cAMP activator, and insulin. The murine preadipocyte cell line, 3T3-L1 cells, are widely used to obtain mature adipocytes *in-vitro* under the stimulation of adipogenesis factors [20]. Following the procedure of transformation initiates, a series of transcriptional factors, including PPAR-γ and C/EBPα, are activated, inducing the expressions of metabolic genes and adipocytokines, such as FABP4, glucose transporter 4 (GLUT4), and leptin [21]. It is reported that after 3T3-L1 cells are stimulated with adipogenesis factors, the epigenetic state of PPAR-γ is regulated by C/EBPα and glucocorticoid receptor [22]. The investigations on histone modification sites at the genomic level show that several functional enhancement sites are observed at approximately 122 kb from the originating site of transcription in *PPAR* [23]. Due to the dynamic changes of histone acetylation during adipogenesis, multiple proteins that regulate these enhancement sites have been proven to be involved in the differentiation process of adipocytes. For example, adipogenesis can be inhibited by suppressing the type II HDAC protein [24]. In the present study, the inhibitory effect of TSA, an inhibitor of HDAC, on adipogenesis and obesity was investigated. We established the adipogenesis *in vitro* model by incubating 3T3-L1 cells with DMI, which was verified by elevated lipid accumulation, increased release of triglyceride and glycerol, upregulated adipocytokines, and promoted expression level of adipocytic transcriptional factors. By the incubation with TSA, these adipogenesis symptoms were significantly reversed, indicating that TSA has a promising inhibitory effect on the transformation from preadipocytes to adipocytes. In addition, in HFD-fed mice, TSA treatment significantly ameliorated the pathological state, elevated body weight and adipose tissue weight, directly indicating the potential therapeutic properties for obesity. In our future work, the inhibitory effect of TSA on the adipocytic transcriptional factors will be verified by co-treatment with the activator of PPAR-γ and C/EBPα.

Adenosine activated protein kinase (AMPK) is an important enzyme involved in the regulation of cellular metabolism. The anabolic enzymes of lipid acids and cholesterol, such as acetyl-CoA carboxylase (ACC) and hydroxymethyl glutarate monoacyl coenzyme A reductase (HMGCR), can be activated by the phosphorylation of AMPK, further decreasing the level of lipid acids and cholesterol in the blood [25, 26]. It is reported that the expression of PPAR-γ and C/EBPα is regulated by the AMPK pathway [27], further contributing to the inhibition of the differentiation from preadipocytes to adipocytes [28]. In the present study, we found that the decreased expression level of p-AMPK induced by DMI treatment was significantly reversed by TSA, indicating an activation effect of TSA against the AMPK signaling pathway. In addition, the inhibitory effect of TSA against the expression of PPAR-γ and the adipogenesis of 3T3-L1 cells was significantly abolished by co-incubation with the inhibitor of AMPK, indicating that the activation of the AMPK pathway was involved in the therapeutic mechanism of TSA. In our future work, the involvement of the AMPK pathway will be further verified by co-administrating the obese mice with TSA and the inhibitor of the AMPK pathway to better understand the therapeutic property of TSA against obesity.

Taken together, our data indicate that TSA inhibited the adipogenesis in 3T3-L1 preadipocytes by activating the AMPK pathway.

## MATERIALS AND METHODS

### Cells and treatments

Mouse embryo 3T3-L1 cells were purchased from ATCC (Maryland, USA) and were cultured in DMEM medium (Thermo Fisher Scientific, USA) containing 10% fetal bovine serum (FBS) (Gibco, USA) at 37° C and 5% CO_2_. For adipogenesis, 3T3-L1 cells were incubated with a differentiation cocktail (DMI) medium (Sigma-Aldrich, USA). Differentiation cocktail (DMI) medium contains 0.5 mM 3-isobutyl-1-methylxanthine (IBMX) (Sigma, USA), 1 μM dexamethasone (Sigma, USA) and 10μg/mL insulin (Sigma, USA).

### Animals and treatments

All animal experiments were performed in accordance with the regulations of the Ethics Committee of Jinan University. The B6 male mice, aged 6-8 weeks were purchased from Beijing Vital River Laboratory Animal Technology Co., Ltd. (Beijing, China), and fed with a high-fat diet (HFD) for 10 weeks consecutively to establish an obesity animal model. The animals were divided into 4 groups: Control (Animals were fed with regular diet), HFD (Animals were fed with HFD and treated with normal saline), 1 mg/kg TSA (Animals were fed with HFD and treated with 1 mg/kg TSA), and 2 mg/kg TSA (Animals were fed with HFD and treated with 2 mg/kg TSA). After the treatments, animals were weighed and sacrificed with euthanasia and the adipose tissues were extracted for histological evaluation and weighing.

### MTT assay

3T3-L1 cells were planted on a 96-well plate (Thermo, Massachusetts, USA) at a density of 5×10^4^/mL, followed by incubation for 4 hours and replacing the medium with a blank DMEM medium. Subsequently, 20 μL MTT (2 mg/mL) solution was added into each well for incubation for an additional 4 hours at 37° C. Formazan crystal pellets were then dissolved with DMSO (Sigma-Aldrich, Missouri, USA). Lastly, the absorbance at 570 nm was measured using a microplate reader (Bio-Rad, California, USA) to calculate the cell viability of each group.

### Oil Red O staining

To visualize the lipid droplets in differentiated 3T3-L1 cells, the Oil-Red-O staining assay (Sigma-Aldrich, Missouri, USA) was performed. Briefly, the cells were fixed with 10% formalin at room temperature for 1 hour, followed by being washed and incubated with an Oil-Red-O working solution for another 1 hour. Subsequently, the cells were washed 3 times with deionized water and the stained lipid droplets were captured. Finally, the calculation of the droplet area was conducted with Gen 5 (Bio Tek, Vermont, USA).

### Measurement of released glycerol

The amount of released glycerol was measured to evaluate lipolysis. In brief, the supernatant of the cells was centrifugated to remove the debris, followed by measuring the concentration of glycerol using a glycerol quantification kit (Biovision, USA) according to the instructions of the manufacturer. Finally, the absorbance at 550 nm was detected with an autoanalyzer (Roche, Basel, Switzerland) to determine the released glycerol.

### The quantification of triglyceride

The cells were washed with PBS followed by centrifugation and resuspended and homogenized in 0.5 mL of 5% NP-40 solution. Subsequently, the samples were heated to 80-100° C for 10 minutes and then cooled down to room temperature. The cells were then centrifugated at a high speed in a microcentrifuge for 2 minutes to remove the insoluble materials. Lastly, the concentration of triglyceride in the samples was detected using a commercial kit (Abcam, Cambridge, UK) according to the instructions of the manufacturer. The microplate reader (Bio-Rad, California, USA) was used to detect the OD values at 570 nm and the concentration of triglyceride was calculated based on the standard curve.

### RT-PCR assay

cDNA synthesis was conducted following isolating total RNAs from the treated cells using the GoScript Reverse Transcription System (Promega, Wisconsin, USA) according to the instruction of the manufacturer. The CFX96 Touch Deep Well Real-Time PCR Detection System (BIO-RAD, California, USA) was used to quantify the relative expression level of the target genes with GAPDH as an endogenous control using the 2^-ΔΔCt^ method. The following primers were used: 5′-tgggaacctggaagcttgtctc-3′ and 5′-gaattccacgcccagtttga-3′ for FABP4; 5′-agaccactcgcattcccttt--3′ and 5′-ccacagactcggcactcaat-3′ for PPARγ; 5′-agcaacgagtaccgggtacg-3′ and 5′-tgtttggctttatctcggctc-3′ for C/EBPα; 5′-ccgagatgtgcgaactgga-3′ and 5′-gaagtcactgtc ttggttgttgatg-3′ for SREBP1C; 5′-agggaggaaaatgtgctgga-3′ and 5′-ggtgaagcccaggaatgaag-3′ for Leptin; 5’-ggagtccactggcgtctt-3’ and 5’-aggctgttgtcatacttctcat-3’ for GAPDH.

### Western blot assay

Cell lysis was extracted from the treated cells utilizing the lysis buffer (Thermo Fisher Scientific, Massachusetts, USA), followed by isolating the total proteins. The proteins were then quantified with the BCA commercial kit (Beyotime Biotechnology, Shanghai, China) and separated using the SDS-PAGE after being loaded. Subsequently, the proteins were transferred to the PVDF membrane, followed by being incubated with primary antibodies against AMPKα (1:2000, Cat#5832, CST, USA), p-AMPKα (1:2000, Cat#5759, CST, USA), and β-actin (1:5000, Cat#sc-47778, SCBT, USA), at 4° C overnight. Lastly, the membrane was incubated with a secondary antibody at room temperature for 1.5 hours and exposed to ECL solution (Beyotime Biotechnology, Shanghai, China). Image J was used to semi-quantify the relative expression level of target proteins.

### Hematoxylin-eosin (HE) staining

The pathological state of adipose tissues was evaluated with HE staining. The adipose tissues were fixed in 4% paraformaldehyde and embedded in paraffin, followed by being cut into 5 μm sections. Subsequently, the sections were dewaxed in xylene and washed with a 100% to 50% ethanol gradient, followed by being stained with hematoxylin (MXB Biotechnologies, Shanghai, China) for 1 minute. Following being differentiated in hydrochloric acid-ethanol for several seconds, the adipose tissues were stained with eosin (MXB Biotechnologies, Shanghai, China) for 5 minutes. Finally, the sections were observed using a biological inverted microscope (Olympus, Tokyo, Japan).

### Statistical analyses

All experiments were repeated at least 3 times. The data are presented as mean ± Standard Deviation (SD), and these data are statistically analyzed by Analysis of Variance (ANOVA) using GraphPad Prism 5.0 software. Statistically significant differences were accepted at P<0.05.
